# The added value of SPECT-CT for the identification of sentinel lymph nodes in early stage oral cancer

**DOI:** 10.1007/s00259-017-3613-8

**Published:** 2017-01-29

**Authors:** Inne J. den Toom, Annelies van Schie, Stijn van Weert, K. Hakki Karagozoglu, Elisabeth Bloemena, Otto S. Hoekstra, Remco de Bree

**Affiliations:** 1 Department of Otolaryngology-Head and Neck Surgery, VU University Medical Center, Amsterdam, The Netherlands; 2 Department of Head and Neck Surgical Oncology, UMC Utrecht Cancer Center, University Medical Center, PO Box 85500, 3508 GA Utrecht, The Netherlands; 3 Department of Radiology and Nuclear Medicine, VU University Medical Center, Amsterdam, The Netherlands; 4 Department of Oral and Maxillofacial Surgery/Oral Pathology, VU University Medical Center/Academic Centre for Dentistry (ACTA) Amsterdam, Amsterdam, The Netherlands; 5 Department of Pathology, VU University Medical Center, Amsterdam, The Netherlands

**Keywords:** Oral cancer, Sentinel lymph nodes, Single-photon emission computed tomography, Lymphoscintigraphy, Lymphatic metastasis

## Abstract

**Purpose:**

To assess the role of single-photon emission computed tomography with computed tomography (SPECT-CT) for the identification of sentinel lymph nodes (SLNs) in patients with early stage (T1–T2) oral cancer and a clinically negative neck (cN0).

**Methods:**

In addition to planar lymphoscintigraphy, SPECT-CT was performed in 66 consecutive patients with early stage oral cancer and a clinically negative neck. The addition of SPECT-CT to planar images was retrospectively analyzed for the number of additional SLNs, more precise localization of SLNs, and importance of anatomical information by a team consisting of a nuclear physician, surgeon, and investigator.

**Results:**

Identification rate for both imaging modalities combined was 98% (65/66). SPECT-CT identified 15 additional SLNs in 14 patients (22%). In 2/15 (13%) of these additional SLNs, the only metastasis was found, resulting in an upstaging rate of 3% (2/65). In 20% of the patients with at least one positive SLN, the only positive SLN was detected due to the addition of SPECT-CT. SPECT-CT was considered to add important anatomical information in two patients (3%). In 5/65 (8%) of the patients initially scored SLNs on planar lymphoscintigrams were scored as non-SLNs when SPECT-CT was added. There were four false-negative SLN biopsy procedures in this cohort.

**Conclusions:**

The addition of SPECT-CT to planar lymphoscintigraphy is recommended for the identification of more (positive) SLNs and better topographical orientation for surgery in sentinel lymph node biopsy for early stage oral cancer.

## Introduction

Sentinel lymph node biopsy (SLNB) in early stage oral cancer is increasingly accepted as standard of care for staging of occult lymph node metastasis. Trials in which only neck dissection is performed after positive SLNB have demonstrated that SLNB is a sensitive method for the detection of occult cervical lymph node metastases. A pooled sensitivity of 91% (95% CI 84–95%) and a negative predictive value of 92–98% were found in a meta-analysis [1], however some lower sensitivity rates had been reported in recent large studies [2, 3]. In most studies, the procedure had a lower accuracy in patients with floor-of-mouth tumors, probably due to the “shine-through phenomenon”; the large injection spot of the primary tumor overshines the eventual sentinel lymph nodes (SLNs) in level I.

Visualization of SLNs is routinely carried out with dynamic and static planar lymphoscintigraphy using a ^99m^Tc-labeled colloidal tracer frequently combined with a blue dye intraoperatively. In our institute, single-photon emission computed tomography with computed tomography (SPECT-CT) is routinely performed. After introduction of the SPECT-CT for SLNB in oral cancer in 2003 by Even-Sapir et al. [4], most studies conclude that SPECT-CT enhances useful information in localization of the SLNs and provides additional SLNs as described in the review of Haerle et al. [5]. Studies of SPECT-CT in SLNB, which included different locations of primary tumors, found especially advantages for tumors with close proximity to the SLN and complex lymphatic regions which is the case in the head-and-neck region [6].

The aim of this present study is to determine the added value of SPECT-CT to the planar dynamic and static lymphoscintigraphic images in patients with early stage oral cancer.

## Materials and methods

From June 2011 until January 2014, 66 consecutive patients with early stage oral cancer and a clinically negative neck (cT1–T2, N0) were retrospectively analyzed. During this period, SLNB was performed as standard procedure in our institution therefore written informed consent was not obtained. All patients underwent transoral excision and SLNB. The SLNB was performed according to the EANM/SENT joint practice guidelines [7]. In this article, we describe only the imaging part of the procedure in our institution in detail, as the entire procedure had been previously described [8].

All patients underwent the procedure in a 2-day protocol with peritumoral injections of ^99m^Tc labeled nanocolloidal albumin (Nanocoll; GE Healthcare, Eindhoven, The Netherlands) in four quadrants at the closest proximity of the primary tumor. The injections had a volume of 0.1–0.2 ml each and the median dose of injected radioactivity was 102 MBq (range, 91–111 MBq). To avoid spillage of the radiocolloid, the patients will be required to perform a mouthwash immediately after injection. No side effects due to the colloidal injections were observed.

Planar and SPECT images were acquired with a SPECT-CT gamma camera (Siemens, Erlangen, Germany). Planar lymphoscintigraphy was started directly after injection of the tracer. Planar images were acquired in dynamic mode (128 × 128 matrix, 20 frames of 1 min) in anteriorposterior projection and static mode (256 × 256 matrix, during 2 min) in anteriorposterior and lateral projections. In addition to the planar imaging, SPECT-CT scans had been routinely performed in all patients without changing the patient’s position. SPECT (matrix 128 × 128, non circular, 32 steps, 40 seconds per step, slice thickness 4.8 mm) took 24 min, CT (40 mAs, 130 kV, slice thickness 1.5 mm) took approximately 5 min.

The SPECT images were reconstructed by filtered back projection (FBP: Generalized Hanning, cut-off 0.90, alpha 0.5, no attenuation correction) and iterative reconstruction (Iterative Flash3D with CT attenuation correction (CTAC): six iterations, eight subsets, Gaussian filter 12). The CT study was reconstructed with 5-mm slice thickness (Kernel B08s) and in soft tissue setting with 2-mm slice thickness (Kernel B30s). Reconstructions were obtained in transversal, sagittal, and coronal planes.

Subsequently, the identified SLNs were anatomically categorized according to the levels of the neck as proposed by the Committee for Head and Neck Surgery and Oncology of the American Academy of Otolaryngology – Head and Neck Surgery (AAO-HNS) [9]. The SLNs were marked on the patient’s skin using a ^57^Cobalt marker and confirmed using a handheld gamma probe (Europrobe II; Eurorad, Strasbourg, France).

In this retrospective analysis, we focused on the additional value of the SPECT-CT imaging on the number of SLNs, their localization, and the additional value of better topographical orientation preoperatively. Exclusion of initially considered SLNs on planar imaging due to SPECT-CT was also considered clinically relevant.

A clear visible and rapidly appearing lymph node was considered to be a SLN according to the definition of Morton [10]. Less visible lymph nodes (especially in presence of a clear SLN) were considered second or third echelon and had not been marked on the skin. In this study, all images were evaluated by a team consisting of a nuclear physician, a head-and-neck surgeon, and an investigator. The team had to reach consensus in every patient. All team members had experience with at least 20 patients with SLNB imaging and early stage oral cancer. First, the planar lymphoscintigraphic images alone were interpreted, thereafter the team compared the planar imaging with the SPECT-CT and the potential additional value had been assessed. Additional hotspots on SPECT-CT that received direct drainage from the primary tumor were considered as SLNs, while level or neck side was not relevant for being an SLN. The additional hot spots found on SPECT-CT were considered also as SLNs if the intensity of uptake in the additional lymph node was at least as hot as considered SLNs on planar lymphoscintigraphy. If the additional hotspots on SPECT-CT were more proximal to the primary tumor compared with other considered SLNs on planar lymphoscintigraphy, they were also scored as SLNs. Additional caudal hotspots with low uptake, not increasing in time, were considered to be second-echelon lymph nodes. A caudal focus with a clearly visible connecting lymphatic vessel from a cranial SLN was also considered a second-echelon lymph node [11]. There was no limit on the number of SLNs.

A calculation of how many SPECT-CT scans are needed to find one additional SLN was also performed, a so-called “number needed to SPECT-CT”. This calculation is a variation on the well-known number needed to treat, which is the inverse of the absolute risk reduction. This number needed to SPECT-CT will be calculated by 100/percentage of patients with (positive) additional SLNs on SPECT-CT.

Anatomical information by SPECT-CT was considered to be important if the head-and-neck surgeon in the scoring team would probably make a different (or more accurate) surgical approach based on the additional information. If regional disease during follow-up occurred after a negative SLNB, the procedure was considered as false negative.

## Results

**Fig. 1:**
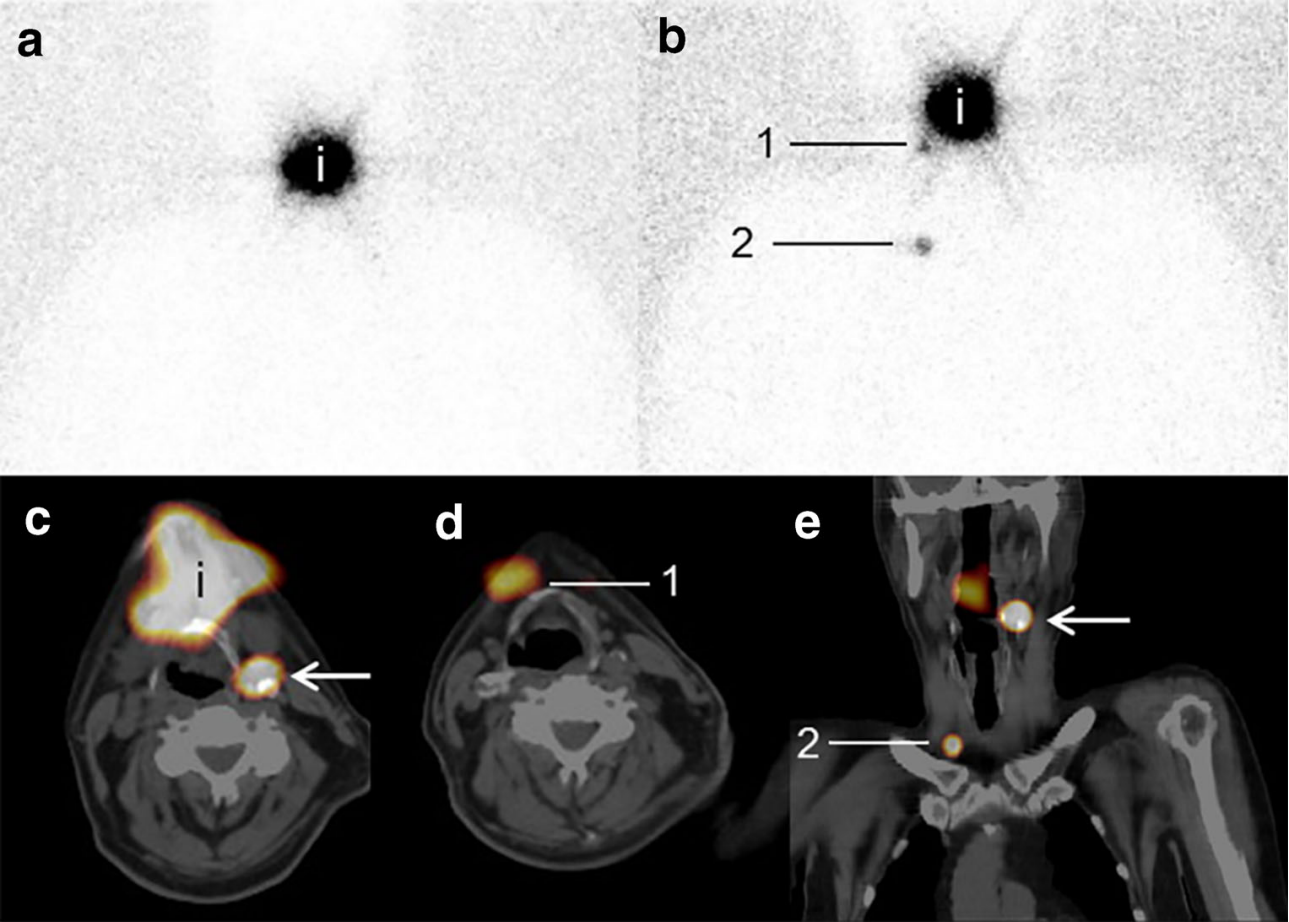
SPECT-CT shows additional SLN level II on the left side (*arrow*). Patient with a clinically T2N0 tongue tumor on the left side. **a** Planar lymphoscintigraphy showed directly post-injection the injection spot (*i*) but no SLNs, 1 h post-injection (**b**) two hotspots, judged as SLN level IA right (*1*) and second echelon lymph node in level IV right (*2*). **c**, **d**, **e** SPECT-CT showed an additional hotspot (*arrow*), considered as SLN level II on the left side. Due to the high amount of uptake in level IV right on SPECT-CT (*2*), exploration with the gamma probe was performed during surgery. During surgery, three SLNs had been identified (level IA right, level IV right, and level II left), all hot, not blue. The SLN level II left contained a macrometastasis. A complementary neck dissection (selective I–IV) had been performed without additional metastasis on histopathological examination. No evidence of disease during follow-up of 32 months was observed

In this cohort of 66 patients, the identification rate of SLNs was 98% (65/66). In one patient, no SLN could be identified on either planar lymphoscintigraphy or SPECT-CT, however this patient showed no metastasis in the untreated neck during regular follow-up for almost 5 years. In 22% (14/65) of the patients, 15 additional SLNs could be identified due to SPECT-CT imaging. The additional SLNs related to other identified SLNs had been found in the same (two SLNs), adjacent (six SLNs) and non-adjacent (four SLNs) levels or in the other neck side (three SLNs). One of these additional SLNs could not be found intraoperatively. In the remaining 14 SLNs, metastases were present in two SLNs (13%). At least one positive SLN was found in ten patients and in two of these patients (20%) the positive SLN had been identified due to the addition of SPECT-CT. These two metastases (one micrometastasis in level III ipsilateral (T1 floor-of-mouth tumor) and one macrometastasis in level II ipsilateral (T2 tongue tumor)) were the only SLNs containing metastasis in the neck, resulting in an upstaging rate of 3% (2/65 patients) (Fig. 1). Five (100/22%) SPECT-CT scans are needed to identify one additional SLN compared with planar lymphoscintigraphy. This “number needed to SPECT-CT” is 34 (100/2.9%) for identification of one additional SLN containing metastasis.

In contrast to these additional SLNs, in 8% (5/65) of the patients, a hot spot was considered to be an SLN based on planar lymphoscintigraphy but was not after SPECT-CT (e.g., injection spot rather than SLN in four patients). In 28% (18/65) of the patients, the anatomic levels of the SLNs on lymphoscintigraphic imaging had been changed with help of the SPECT-CT imaging. In one patient, SPECT-CT identified one additional SLN, but also one considered SLN on planar lymphoscintigraphy could be scored as non-SLN. This results in a full concordance rate according to the number and level of SLNs between planar lymphoscintigraphy and SPECT-CT imaging of 54% (35/65).

With respect to the location of the primary tumor, SPECT-CT identified more additional SLNs in patients with floor-of-mouth tumors compared with tumors of the tongue (42 vs. 13%, *p* = 0.07). In both tumor subsites, one additional SLN showed metastasis and in each of these two tumor subsites SLNB was considered as false negative in two patients (Table 1).

**Table 1:** Additional SLNs due to SPECT-CT imaging according to tumor localization

Location	All patients	Patients with additional SLNs	Positive additional SLNs	False negatives
Tongue	39	5 (13%)	1	2
Floor of mouth	19	8 (42%)	1	2
Buccal mucosa	4	1 (25%)	0	0
Other	3	0	0	0
Total	65	14 (22%)	2	4

*SLNs* sentinel lymph nodes

**Fig. 2:**
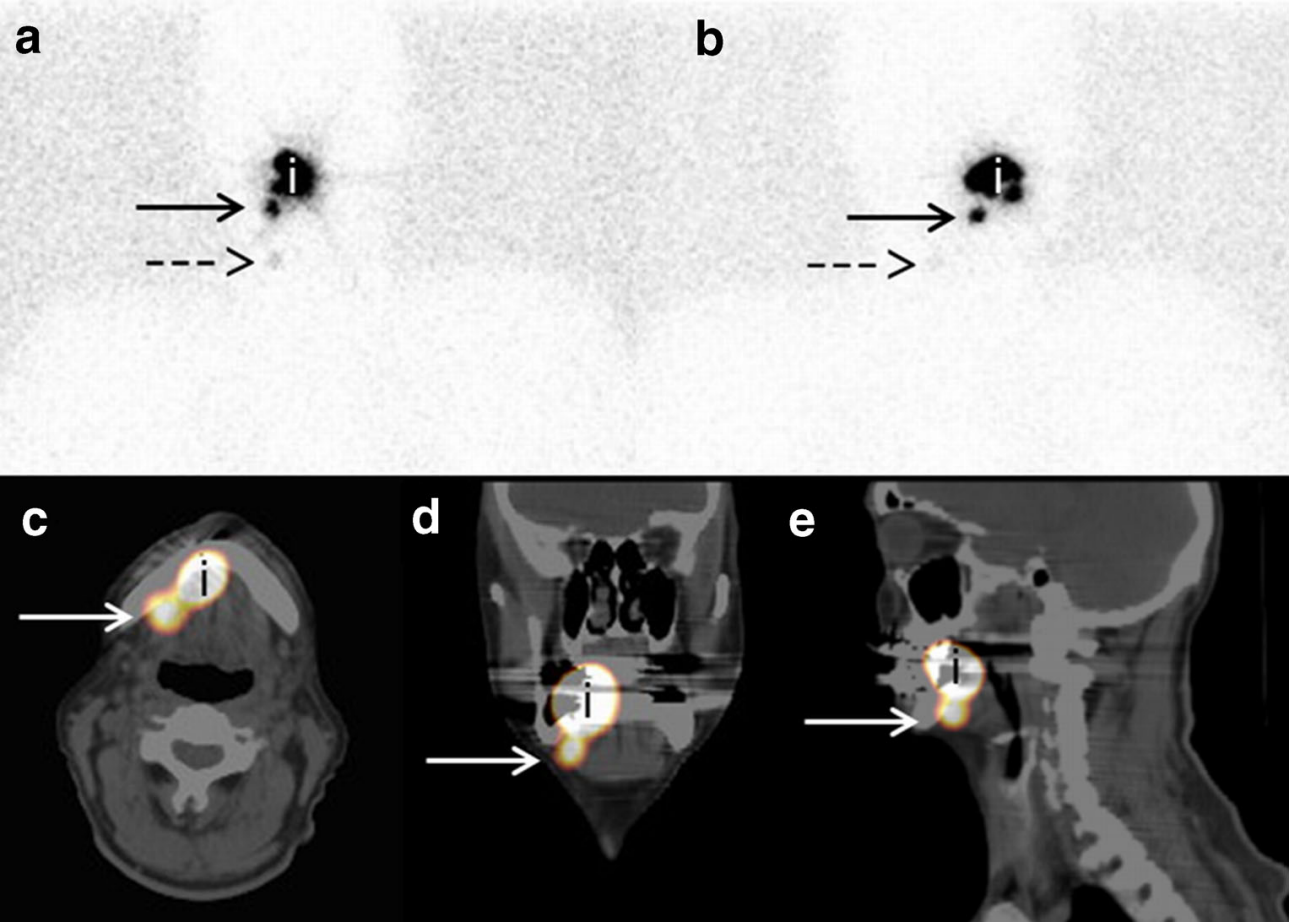
Example of better topographical orientation. Patient with a clinically T2N0 floor-of-mouth tumor on the right side. **a** On the planar lymphoscintigraphy, the hotspot is clearly visible (*arrow*), considered to be a sentinel lymph node in level I. Also, a less-visible hot spot was observed (*dashed arrow*), considered to be a second echelon node. **b** Lateral projection of the planar lymphoscintigraphy on the right side of the neck with the same intense hotspot in level I (*arrow*) and very weak uptake in the considered second echelon node (*dashed arrow*). **c**, **d**, **e** SPECT-CT shows a hotspot just behind the mandible (*white arrow*) with close relationship to the injection spot of the primary tumor (*i*) and was actually considered as a sublingual node

Important additional anatomical information of the SLNs preoperatively due to SPECT-CT imaging was observed in 3% of the patients (2/65), making a more accurate surgical approach possible (Fig. 2). Obviously, in the rest of the patients, a better topographical orientation for the surgeon had been provided by SPECT-CT compared with planar imaging, but the reading team had the impression that SLNB could also be successfully performed with planar lymphoscintigraphy only.

## Discussion

In this study of 66 patients with early stage oral cancer, we retrospectively evaluated the additional value of SPECT-CT compared to the conventional planar lymphoscintigraphy for the detection of SLNs. To our knowledge, this is the largest single-center study investigating the additional value of SPECT-CT in oral cancer. In a multidisciplinary setting, both imaging modalities were separately investigated, and in 22% of the patients, additional SLNs were found on SPECT-CT imaging. In 20% of the SLN-positive patients, the positive SLN had been identified only with SPECT-CT. These additional positive SLNs result in an upstaging rate to a positive neck of 3% in the total cohort, in other words we have to make 34 SPECT-CT scans to identify one additional positive SLN. These results are more or less comparable with some previously reported studies [12–15], but some other studies report higher rates [16, 17]. A study with barely no additional SLNs due to SPECT-CT had also been reported [18]. It is hard to find a reasonable explanation for these differences in almost comparable patient groups and comparable imaging modalities [5]. One reason may be the difference in imaging protocols throughout Europe with respect to the amount of injected radioactivity and the time of injection related to the surgical procedure (same- or 2-day protocols) [19]. Another explanation can be the practice variation in defining SLNs on planar lymphoscintigraphy as shown by Flach et al. [20]. In order to perform consistent lymphoscintigraphic evaluation, defining the SLNB concept is essential. There are many definitions of the SLN and many articles discuss the subject. The definition of Morton et al. [10], which says ‘a sentinel node is the first draining lymph node on the direct lymphatic drainage pathway from the primary tumor site’ best reflects the stepwise spread of cancer through the lymphatic system. However, this is a theoretical concept, and does not always aid the clinician in interpreting a lymphoscintigraphic scan as an individual situation, because it is regularly not so clear-cut as this theory. Describing how to interpret lymphoscintigraphic imaging with a view to identify foci (hot spots) as SLN in a simple and straightforward way is not easy. In a study on interobserver agreement, many experienced observers correctly considered SLNs as the lymph nodes directly draining from the injection site, and/or single radioactive nodes in a basin, whereas other important criteria as uptake intensity, time of appearance, relevance of neck side and level were rated differently. Interobserver agreement can be influenced by a number of factors. If a single focus is visualized, there will be no disagreement. However, in a complex nodal basin as the neck area, several foci are often visible. This harbors an increased risk of not identifying the correct SLN and/or misinterpretation of second echelon nodes as SLNs [20]. In view of the literature, it seems that despite the additional information, SPECT-CT is not yet able to solve the problem of difficult interpretation of SLNs.

The study of Haerle et al. [13] showed all their additional SLNs in the same or adjacent levels as hotspots detected by planar lymphoscintigraphy alone and they suggest that even necks without hotpots should be explored with the gamma probe intraoperatively, based on the fact that the gamma probe identified SLNs in patients without hotpots on imaging. In contrast to their study, we found seven additional SLNs in a non-adjacent level or even in the other neck side compared to planar lymphoscintigraphy. However, we still found the (dynamic) planar lymphoscintigraphy of additional value in differentiating SLNs and second-echelon nodes, especially using the criterion of rapidly emerging hot spots. Therefore, we recommend a combination of planar static and dynamic imaging followed by SPECT-CT as the currently best imaging procedure for SLNB.

We hypothesized that we could find additional SLNs due to SPECT-CT, especially in patients with SLNs in close proximity to the primary tumor, as is the case for SLNs in level I with a primary tumor in the floor of mouth. Indeed, in five patients, additional SLNs had been identified in level I; however, four of these patients had a tongue tumor and only one had a floor-of-mouth tumor. In addition, in four patients (two tongue tumors, two floor-of-mouth tumors) a hot spot considered to be a SLN could be identified as injection spot rather than SLN in level I by SPECT-CT. We found a trend for more additional SLNs in floor-of-mouth tumors compared with tongue tumors, also resulting in a lower number needed to SPECT-CT (not presented).

Despite our experience with SLNB in oral cancer, we report a relatively high number of false-negative patients in this study. In this small cohort of 19 floor-of-mouth tumors, two false-negatives were present, compared to two false-negatives in 39 tongue tumors. In one patient with a left-sided floor-of-mouth tumor, the initially found SLN was located in level I on the right side; then this patient returned with a metastasis in level I on the left side 6 months after SLNB, which had been probably missed on the planar lymphoscintigraphy and SPECT-CT. The other false-negative patient with a floor-of-mouth tumor had a regional metastasis in level III 13 months after SLNB. Both patients are alive with no evidence of disease for more than 2.5 years. Both patients with a tongue tumor and false-negative SLNB had regional metastasis in level II ipsilateral, approximately 1 year after SLNB. One patient is alive with no evidence of disease for 3 years, and one patient was lost to follow-up.

In our opinion, SPECT-CT did not solve the problems of the lower accuracy in patients with floor-of-mouth tumors, despite the higher number of additional identified SLNs due to SPECT-CT. The finding that additional SLNs were mainly found in other levels than level I suggests that the “shine through phenomenon” remains the most common problem in floor-of-mouth tumors. Other new technologies and procedures, e.g., superselective neck dissection of level I, ^99m^Tc-tilmanocept, fluorescence-guided SLNB, and PET/CT lymphoscintigraphy with ^89^Zr-nanocolloidal, seem promising to improve the accuracy of the SLNB in floor-of-mouth tumors [21–25].

It should be clear that SPECT-CT allows better anatomical information for the surgeon preoperatively in all cases. In two patients, our team had determined this information of evident importance. We described a sublingual node on SPECT-CT, which had been scored as level I on planar lymphoscintigraphy (Fig. 2). Sieira-Gil et al. [15] had also found sublingual SLNs by SPECT-CT (two cases), which had not been detected by planar lymphoscintigraphy. Due to the better topographical orientation, the anatomical levels of the SLNs had been changed in 28% of the patients and better delineation against surrounding tissues could be done. Nevertheless, it still remains difficult to determine the extent to which SPECT-CT influences the surgical approach related to planar lymphoscintigraphy particularly due to the use of the handheld gamma probe just before incision. To get more insight in this additional value, the surgical procedure should be planned blinded to the results of the SPECT-CT and replanned after revealing the SPECT-CT. Our study suggests that SPECT-CT is helpful preoperatively and probably because of the better anatomical orientation surgery could be performed more safely than with planar lymphoscintigraphy alone.

We report a relatively low concordance rate of 54% between planar lymphoscintigraphy and SPECT-CT in comparison to the concordance rate of 81% of Haerle et al. [13]. However, they only report a concordance rate according to number of hotpots on both imaging modalities, where we also include changes of anatomical levels of the hotspots in this rate.

Nowadays, SLNB for early stage oral cancer is gaining more acceptance worldwide and has recently been included in many guidelines. In the beginning, SLNB had been reported to be safe with planar lymphoscintigraphy alone, but, in general, all studies published in the last 5 years had performed SLNB with SPECT-CT in addition to planar imaging despite only moderate evidence in reported literature so far.

We conclude that SPECT-CT after static and dynamic planar lymphoscintigraphic imaging has the potential to detect more (22%) SLNs than planar lymphoscintigraphy alone, especially in patients with floor-of-mouth tumors, resulting in an upstaging rate of 3% in all patients. In 20% of the patients with at least one positive SLN, the only positive SLN was detected due to the addition of SPECT-CT. Moreover, SPECT-CT provides better topographical orientation for the surgeon preoperatively. We recommend the addition of SPECT-CT in SLNB for patients with early stage oral cancer, however other improvements are still mandatory to increase the accuracy of this procedure.
